# Endotyping in ARDS: one step forward in precision medicine

**DOI:** 10.1186/s40001-024-01876-7

**Published:** 2024-05-14

**Authors:** Andréanne Côté, Chel Hee Lee, Sayed M. Metwaly, Christopher J. Doig, Graciela Andonegui, Bryan G. Yipp, Ken Kuljit S. Parhar, Brent W. Winston

**Affiliations:** 1 Department of Medicine, Institut Universitaire de Cardiologie et de Pneumologie de Quebec-Université Laval, Quebec, Canada; 2 Department of Critical Care Medicine, Medicine and Biochemistry and Molecular Biology, Health Research Innovation Center (HRIC), University of Calgary, Room 4C64, 3280 Hospital Drive N.W., Calgary, AB T2N 4Z6 Canada; 3 Department of Mathematics and Statistics, University of Calgary, Calgary, Canada; 4 School of Medicine, Medical Sciences and Nutrition, University of Aberdeen, Aberdeen, UK; 5 Division of Internal Medicine, Aberdeen Royal Infirmary, NHS Scotland, Aberdeen, UK; 6 Depatments of Medicine, University of Calgary, Calgary, Canada; 7 Department of Biochemistry and Molecular Biology, University of Calgary, Calgary, Canada

**Keywords:** Acute respiratory distress syndrome, Cluster analysis, Mixed data analysis, Feature selection

## Abstract

**Background:**

The Berlin definition of acute respiratory distress syndrome (ARDS) includes only clinical characteristics. Understanding unique patient pathobiology may allow personalized treatment. We aimed to define and describe ARDS phenotypes/endotypes combining clinical and pathophysiologic parameters from a Canadian ARDS cohort.

**Methods:**

A cohort of adult ARDS patients from multiple sites in Calgary, Canada, had plasma cytokine levels and clinical parameters measured in the first 24 h of ICU admission. We used a latent class model (LCM) to group the patients into several ARDS subgroups and identified the features differentiating those subgroups. We then discuss the subgroup effect on 30 day mortality.

**Results:**

The LCM suggested three subgroups (*n*_1_ = 64, *n*_2_ = 86, and *n*_3_ = 30), and 23 out of 69 features made these subgroups distinct. The top five discriminating features were IL-8, IL-6, IL-10, TNF-a, and serum lactate. Mortality distinctively varied between subgroups. Individual clinical characteristics within the subgroup associated with mortality included mean PaO_2_/FiO_2_ ratio, pneumonia, platelet count, and bicarbonate negatively associated with mortality, while lactate, creatinine, shock, chronic kidney disease, vasopressor/ionotropic use, low GCS at admission, and sepsis were positively associated. IL-8 and Apache II were individual markers strongly associated with mortality (Area Under the Curve = 0.84).

**Perspective:**

ARDS subgrouping using biomarkers and clinical characteristics is useful for categorizing a heterogeneous condition into several homogenous patient groups. This study found three ARDS subgroups using LCM; each subgroup has a different level of mortality. This model may also apply to developing further trial design, prognostication, and treatment selection.

**Supplementary Information:**

The online version contains supplementary material available at 10.1186/s40001-024-01876-7.

## Introduction

Acute respiratory distress syndrome (ARDS) is a common clinical condition in the intensive care unit (ICU) and a significant cause of morbidity and mortality [1–3]. ARDS represents 10.4% of all ICU admissions worldwide, affecting 23.4% of all patients requiring mechanical ventilation [4]. ARDS-related mortality before ICU discharge has been estimated at 35.3% overall, including 29.7, 35.0, and 42.9% for mild, moderate, and severe diseases, respectively. Despite ARDS's clinical, societal, and economic burden, there is no specific therapy, and the mainstay of management is supportive care [5].

ARDS is an acute onset of non-cardiogenic pulmonary edema, bilateral pulmonary infiltrates, and hypoxemia [5, 6]. The definition, including the most recent consensus nominally known as the Berlin criteria, describes clinical characteristics without considering the pathophysiological processes leading to lung injury. Although pneumonia and sepsis are common causes, ARDS is a complex heterogeneous syndrome [5, 7, 8]. The most recent example of the heterogeneity of ARDS is COVID-19. Although COVID-19 patients have oxygenation and radiographic characteristics that meet the clinical criteria of ARDS, many other parameters (such as ventilatory mechanics and inflammatory mediator profiles) appear to be different [9, 10]. In addition, despite a common etiologic cause, more than one disease subtype has been described. Although ARDS has heterogeneous causes and manifestations, diffuse alveolar damage (DAD) is the histologic hallmark [7]. However, using accepted pathologic criteria [11, 12], DAD is identified in approximately half of the biopsy samples from patients diagnosed with ARDS [3]. Other investigators have suggested ways of subdividing the disease [13–15] to improve the identification of patients at risk for ARDS, improve prognostication, develop targeted therapy, and inform clinical trial design [16].

Endotyping is one approach to stratify patients. Biomarkers are an attractive tool for identifying different ARDS subtypes and have been a focus of study in the past decade [4]. Therapy-directed genotyping using circulating biomarkers has proven to be an emerging strategy for targeted oncologic therapy but is infrequently utilized in critical care, specifically in ARDS-based research. Recent studies regrouping clinical characteristics and biomarkers have identified two distinct biological subgroups of ARDS [15, 17, 18]. These subgroups appear to be associated with explaining differential outcomes when applied in retrospective studies. These early studies offer promise for the potential of genotyping and studying potential mechanisms of ARDS. However, studies to date have not included patients from heterogeneous regions. Our study aimed to identify clusters in a Canadian-based ARDS patient population using a combination of clinical characteristics and blood biomarkers, examine any association with mortality, and describe similarities or differences with prior published ARDS subgroups.

## Methods

### Study design

This is an observational, cross-sectional study of adult patients (> 17 years of age) with ARDS entered into the Critical Care Epidemiologic and Biologic Tissue Resource (CCEPTR) tissue bank at the University of Calgary. Written informed consent was obtained from each subject and/or their legal surrogates before data collection and sample storage according to the Conjoint Health Research Ethics Board of the University of Calgary, REB15-0348_MOD5).

### Description of the cohort

The samples and clinical data were collected from adult subjects (> 17 years of age) following ICU admission for suspected infection/sepsis at Foothills Medical Centre or Peter Lougheed Centre; both tertiary care academic multisystem intensive care units in Calgary, Alberta, Canada.

Patients were identified as having ARDS and included in the study if they were on mechanical ventilation on the first day of their ICU stay, had PaO_2_/FiO_2_ ratios ≤ 300 and had a chest X-ray confirming alveolar infiltrates in more than one quadrant. Patients were excluded if they had one of (1) a diagnosis of congestive heart failure (CHF) defined by ejection fraction (E.F.) < 40% on echocardiography or the attending team gave a diagnosis of CHF, (2) if they were immunocompromised, or (3) if the patient died within 24 h of study enrollment.

### Clinical data collection

Clinical data on all patients were extracted from an ICU-specific integrated bedside clinical information system (Metavision, iMDsoft, Tel Aviv Israel), which prospectively captured clinical demographic and physiologic devices data, including ventilation parameters and measures, laboratory results, and outcome data. We have previously validated this information system by manual audit as a reliable data source for quality improvement and research. Fifty total potential clinical covariates were identified, including 13 risk factors, 12 comorbidities, 10 clinical laboratory results, eight non-respiratory clinical measures, and seven ventilatory parameters.

### Biomarker selection and assay procedures

A priori, we identified 12 biomarkers for analysis. The biomarkers were selected considering four broad criteria: (1) are a potential measure of lung epithelial injury (e.g., RAGE), (2) are a marker of inflammatory injury including endothelial injury [e.g., plasminogen activator inhibitor-1 (PAI-1)], (3) assay validated in our laboratory, and/or (4) have been identified in prior work of interest in identifying hyperinflammatory or hypo-inflammatory endotypes in ARDS [14]. Plasma samples were collected within 24 h of ICU admission/ARDS diagnosis. Samples were aliquoted into 250 µl aliquots and frozen at − 80 °C for single use [19]. We measured protein C (P.C.) antigen levels quantified by a sandwich-style ELISA from plasma samples using a matched-pair antibody set (Affinity Biologicals, Ancaster, ON, Canada). The levels of the remaining biomarkers were measured by electrochemiluminescence technology using a Meso QuickPlex SQ 120 instrument (Meso Scale Discovery) equipped with Discovery Workbench 4.0 software for data acquisition and analysis.

### Variables available for analysis

The variables entered in the model were chosen based on the presence of at least one of the following: (1) are routinely measured in a clinical setting, (2) are biomarkers previously identified as having a putative role in ARDS pathophysiology, and (3) represented a distinguishing feature of ARDS.

### Statistical analysis

The baseline characteristics are described using descriptive statistics with measures of central tendency (median) and dispersion (interquartile range, IQR) for continuous variables and counts and percentages for categorical variables. Continuous variables with more than 20% missing values were excluded, and the MICE package was used to impute missing values. They were then transformed into a logarithm scale and standardized with mean zero and unit variance. Risk factors and comorbidity conditions with a single level were also excluded. Binary indicators for risk factors and comorbidity conditions coded positively in less than 10% of cases were removed. We also excluded highly collinear features whose Pearson correlation coefficient was greater than 0.9. Finally, functional variables were excluded. For example, APACHE II was collected as a clinical descriptor but not included in analytical models as it is derived from the other clinical variables. After following the exclusion criteria, 51 variables (33 continuous and 18 discrete variables) were available in the study, and they are listed in Table 1. In the table, we have included variables with missing values and those that underwent imputation for information.

**Table 1:** Descriptive summary of all baseline characteristics used as input for clustering analysis—Clinical variables, comorbidities, risk factors, mechanical ventilation, lab results, biomarkers, and endpoints

Clinical variables	*N* = 180	Nmiss	After imputation
Age (years)	59.50 [49.75, 71.00]	0	
Female sex (%)	74 (41.1%)	0	
BMI (kg/m^2^)	29.0 [24.78, 34.10]	24	28.90 [25.40, 34.13]
Heart rate (beats/minute)	100.0 [82.50, 113.5]	5	100.0 [85.25, 114.0]
APACHE II	22 [18, 28]	7	NA
GCS	49 (27.2%)	0	
Vasopressor use	143 (79.4%)	0	
Prone	4 (2.2%)	0	
Ionotrope	33 (18.3%)	0	
Comorbidity			
Alcohol use disorder	46 (25.6%)	0	
Cardiovascular accident	12 (6.7%)	0	
Chronic kidney disease	23 (12.8%)	0	
Chronic liver disease	21 (11.7%)	0	
Chronic obstructive pulmonary disease	45 (25.0%)	0	
Coronary arterial disease	38 (21.1%)	0	
Diabetes	44 (24.4%)	0	
Heart failure	22 (12.2%)	0	
ARDS trigger			
Aspiration	18 (10.0%)	0	
Hypovolemic or distributive (not sepsis) shock	76 (42.2%)	0	
Pneumonia	110 (61.1%)	0	
Sepsis	154 (85.6%)	0	
Surgery abdominal	45 (25%)	0	
Mechanical ventilation			
Respiratory rate (breaths/min)	22 [15, 25]	8	22 [15, 25]
PF ratio	172.5 [96, 235.8]	0	
Plateau pressure (cm H_2_O)	27 [22, 31]	6	27 [22, 31]
PEEP (cm H_2_O)	10 [8.0, 12]	10	10 [8, 12]
Tidal volume (mL)	540 [460, 610]	11	544 [467.3, 613.5]
Minute ventilation (L/min)	12 [10, 13]	10	12 [10, 13]
Lab results			
Blood glucose (mmol/L)	7.70 [6.30, 9.45]	1	7.7 [6.30, 9.43]
Serum lactate (mmol/L)	1.7 [1.2, 3.3]	1	1.7 [1.2, 3.3]
White blood cell count (× 10^9^/L)	12.90 [7.8, 19.70]	5	12.85 [7.68, 19.65]
Platelet count (× 10^9^/L)	181 [116, 251]	6	183 [116, 245]
Hematocrit	0.33 [0.29, 0.38]	0	
Sodium (mmol/L)	139 [137, 143]	0	
PaCO2 (mmHg)	37 [32, 44]	0	
Bicarbonate (mmol/L)	20 [16, 23]	0	
Creatinine (µmol/L)	97.5 [66.8, 181.0]	4	97.5 [66.75, 175.0]
Biomarker			
Protein C (IU/ml)	63.17 [45.00, 87.56]	3	63.26 [44.81, 87.63]
IL-6 (pg/ml)	152.6 [30.29, 1072]	4	141.0 [30.01, 1072]
IL-8 (pg/ml)	61.17 [19.25, 286.3]	4	61.17 [18.97, 286.3]
IL-10 (pg/ml)	7.05 [2.24, 20.00]	4	6.92 [2.24, 20.0]
TNF-α (pg/ml)	8.220 [4.358, 19.27]	4	8.27 [4.45, 18.75]
ANG-2 (ng/ml)	23750 [14520, 54630]	4	23300 [13810, 54630]
RAGE (pg/ml)	1112 [509.4, 2602]	4	1062 [491.2, 2528]
vWF (pg/ml)	543400 [184200, 1399000]	4	546900 [184200, 1429000]
TNF-R1 (ng/ml)	17660 [8400, 27560]	4	17660 [8291, 28560]
ICAM-1 (ng/ml)	791600 [553100, 1121000]	4	788700 [540900, 1101000]
PAI-1 (ng/ml)	119500 [50460, 382100]	4	119500 [50460, 367500]
SPD (ng/ml)	3313 [1446, 7065]	4	3313 [1512, 7227]
EndPoint			
ICU mortality	44 (24%)	0	
ICU length of stay (Days)	10.0 [6.0, 18.8]	0	
Days of ventilation	8.00 [5.00, 14.00]	0	

Data are reported by frequency and proportion (%) for discrete variables and median and interquartile range [Q1, Q3] for continuous variables

*APACHE* Acute Physiology and Chronic Health Evaluation, *BMI* body mass index, *ICU* Intensive Care Unit, *PF* ratio of partial pressure of arterial oxygen to inspired oxygen percentage, *PEEP* positive end-expiratory pressure, *PaCO2* partial pressure of arterial carbon dioxide, *IL* interleukin, *TNF-α* tumor necrosis factor α, *ANG-2* Angiopoietin 2, *RAGE* receptor of advanced glycation end products, *vWF* von Willelbrand factor, *TNF-R1* tumor necrosis factor receptor 1, *ICAM-1* intercellular adhesion molecule-1, *PAI-1* plasminogen activator inhibitor-1, *SPD* surfactant protein D and *Prot C* Protein C, *Nmis* the number of missing values per variable before imputation, After imputation summary statistics after imputation per variable

A latent class model (LCM) proposed by Marbec et al. was employed with the VarSelLCM package [20, 21] since this model permits cluster analysis with mixed-type data and simultaneously identifies the most discriminative variables. In addition, the model supports two scenarios when the number of variables is smaller or larger than the number of samples. The Bayesian information criterion (BIC) and maximum integrated complete-data likelihood (MICL) criterion were utilized in choosing the optimal number of clusters. A discriminating power index ranked the input variables. A Kaplan–Meier estimator was employed to explore the difference in 30 day mortality by the clusters found by LCM. We also calculated the information value (IV) to rank the input feature in terms of the importance of predicting mortality, using 0.3 as a threshold of clinical association [22]. All analyses were carried out using standard statistical software, R-4.0.0, with the packages VarSelLCM, survival, stats, MASS, and Information.

To compare our analysis and model with previous models, we cross-classified our clustering results to the hyper- and hypo-inflammatory subphenotypes suggested by Sinha et al. (2020) [18] and this is presented in the results.

## Results

### Characteristics of study cohort

Two hundred eight patients were identified with a PaO_2_/FiO_2_ (P/F) ratio of less than 300, of which 28 patients were excluded as 14 had chest X-rays that did not have more than one quadrant of disease, nine were not receiving mechanical ventilatory support, and five were misclassified (CHF was present or suspected).

A description of the demographics, clinical, and biomarker characteristics of the study cohort is shown in Table 1. 58.9% were male, with a median age [IQR] of 60 [51, 71]. The most common contributing cause of ARDS was sepsis (85.6%), of which 29.6% (44/154) was from a pulmonary source. Aspiration was the cause in 10% of patients, transfusion-associated lung injury in 6.1%, pancreatitis in 3.9%, and shock associated with trauma in 1.7%. Many patients had comorbidities, the most common including chronic respiratory disease, coronary atherosclerosis, alcohol use disorder, and diabetes. Twenty-two patients (12.2%) had a prior heart failure history (without evidence of acute hydrostatic pulmonary edema on this ICU admission). The median Apache II score was 22 [18, 28]. Vasopressors were used for 79.4% of the patients. The median P/F ratio was 170 [96, 240]. Ventilatory parameters included a median PEEP value of 10 [8, 12] cm H_2_O with a median tidal volume of 590 mL [520, 630] for men and 480 mL[430, 520] for women, and the median plateau was 26 cm H_2_0 [22, 31]. The median lactate was 1.7 mmol/L [1.2, 3.3], with a white blood cell count of 12.9 [7.8, 19.7] and a platelet count of 181 [116, 251] and serum creatinine of 98 mmol/L [67, 180]. Forty-four (24%) of patients died in ICU. The median ICU length of stay was 10 days [6, 18.3].

### Cluster analysis for subgroup identification

**Fig. 1:**
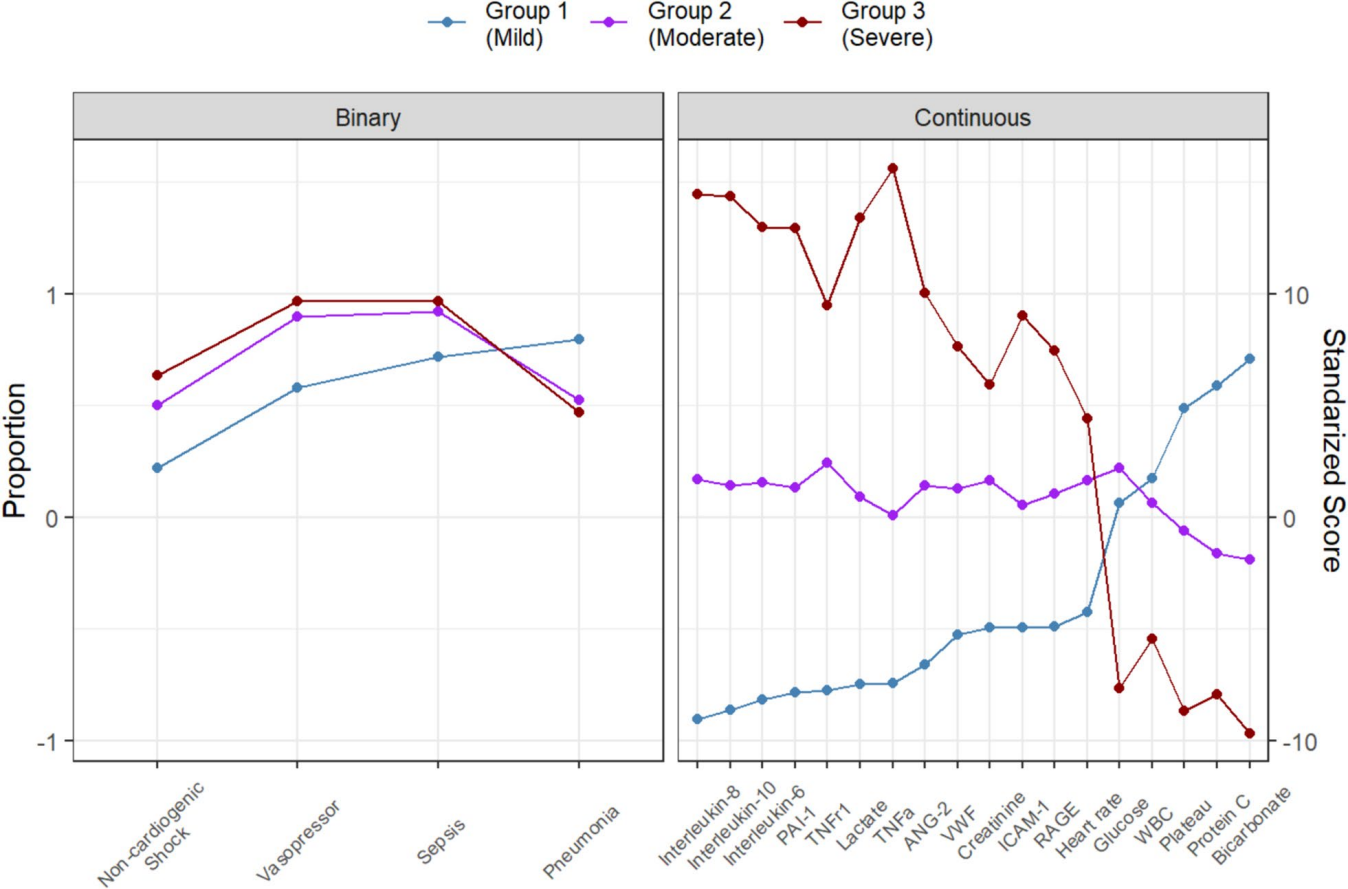
Differences in a standardized score of each continuous variable and proportion of each binary variable by subtype. In terms of mortality, subtypes are referred to as mild, moderate, and severe. Variables are sorted in ascending order in the mild group. Note that *IL* interleukin, *TNF-α* tumor necrosis factor α, *ANG-2* Angiopoietin 2, *RAGE* receptor of advanced glycation end products, *vWF* von Willelbrand factor, *TNF-R1* tumor necrosis factor receptor 1, *ICAM-1* intercellular adhesion molecule-1, *PAI-1* plasminogen activator inhibitor-1, *SPD* surfactant protein D and *Prot C* Protein C

**Fig. 2:**
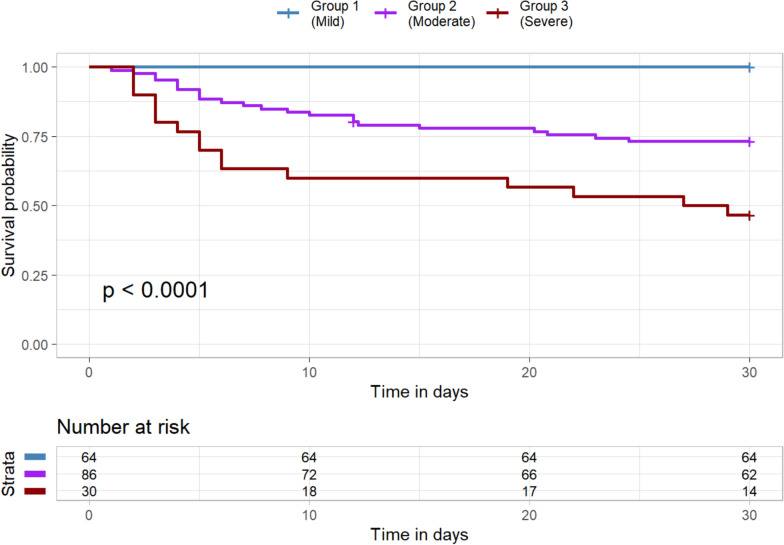
Kaplan–Meier estimate of 30-day patient survival with log-rank test p-value by subgroup

**Fig. 3:**
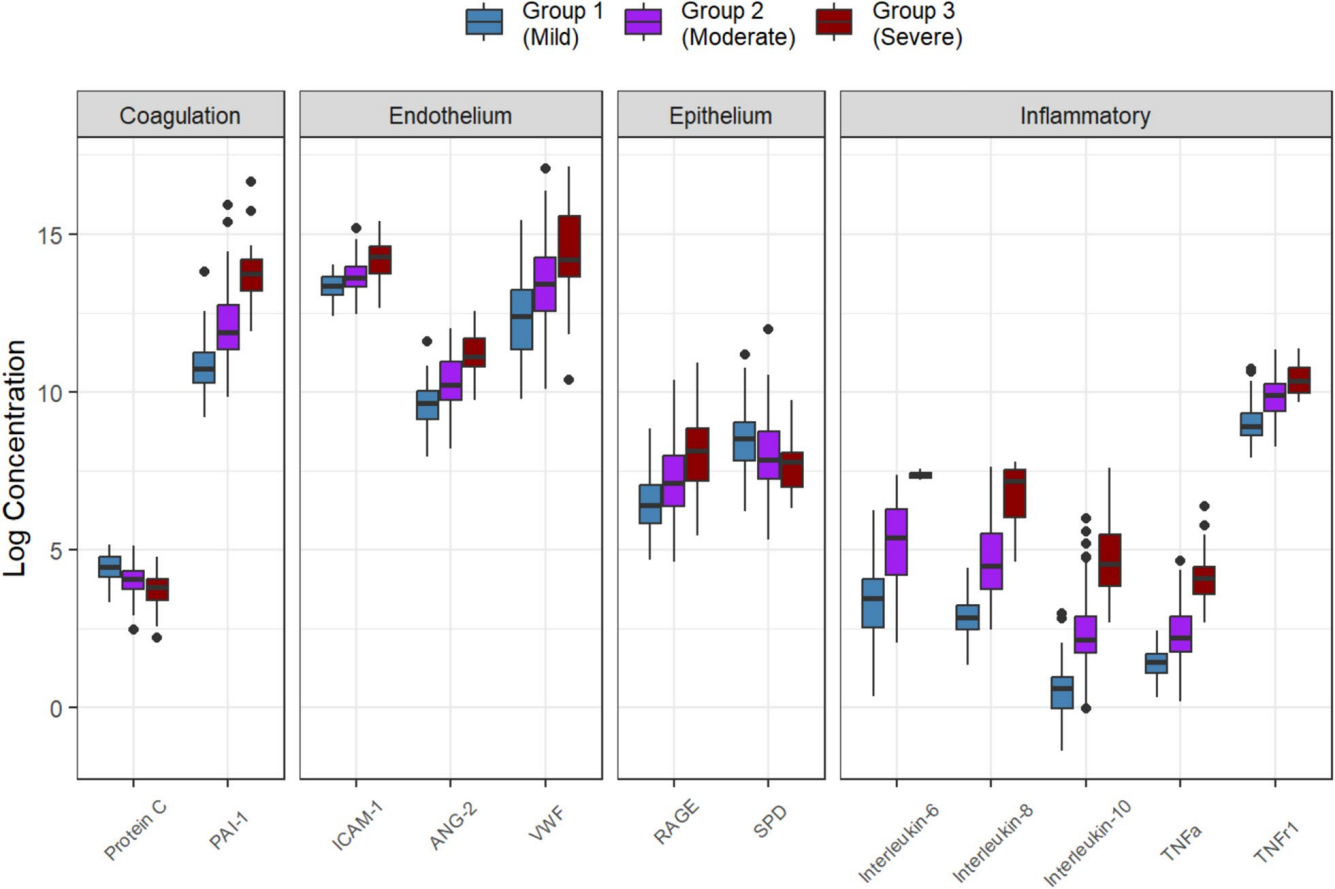
Side-by-side boxplot of cytokine concentration in logarithm scale by subgroup. Note that *IL* interleukin, *TNF-α* tumour necrosis factor α, *ANG-2* Angiopoietin 2, *RAGE* receptor of advanced glycation end products, *vWF* von Willelbrand factor, *TNF-R1* tumour necrosis factor receptor 1, *ICAM-1* intercellular adhesion molecule-1, *PAI-1* plasminogen activator inhibitor-1, *SPD* surfactant protein D and *Prot C* Protein C

We fitted a model to the data by changing the number of subgroups. The model with three subgroups shows the maximum BIC and MICL values (as shown in Table S1 and Figure S1). Twenty-three variables were selected as discriminatory from this model: 8 were clinical measures, 3 were risk factors, and 11 were biomarkers (Table 2). Patients in Group 1 (Group 1 vs. Total) appeared different across multiple variables. For example, the heart rate [median] was lower [88 vs. 100], vasopressors were used less frequently (57.1 vs. 79.4%), had better P/F ratios (197.0 vs. 172.5), lower serum lactate levels (1.10 vs. 1.7), higher platelet counts (236 vs. 181) and less kidney injury as measured by serum creatinine. Endotype subgroup 1 had evidence of lower biomarkers, including lower inflammatory markers (e.g., TNFa, TNF-R1, Il-6, IL-8), lower anti-inflammatory markers (e.g., Il-10), and lower endothelial/coagulation biomarkers (e.g., vWF, ICAM-1, PAI-1), as shown in Fig. 1. When considered in isolation, RAGE, a lung epithelial biomarker, was comparable across sub-groups. When variables with binary results were only evaluated in the LCM, only two subgroups were evident; however, when continuous variables were also considered, there was an evident difference with three subgroups that appeared (Fig. 2). The three endotypes are distinct in terms of the risk of ICU-associated mortality, with Group 1 having no mortality (0%), ICU Group 2 having a 29% ICU mortality, and Group 3 having a 63% mortality before ICU discharge; Group 3 had an earlier and higher rate of death (Fig. 3).

**Table 2:** Sub-group characteristics of the features found by the latent class modeling approach. Score = the value of information criterion, MICL, for clustering

	Name	Score	Group 1 (Mild)	Group 2 (Moderate)	Group 3 (Severe)	P-value
			N = 64	N = 86	N = 30	
Clinical observation						
1	Heart rate (beats/min)	3.02	88.50 [74.50, 101.5]	104.5 [90.75, 116.25]	111.0 [99.00, 122.0]	< 0.001
2	Vasopressor use	11.64	37.0 (57.8%)	77.0 (89.5%)	29.0 (96.7%)	< 0.001
Lab results						
3	Glycemia (mmol/L)	4.16	7.50 [6.50, 9.20]	8.25 [6.43, 10.30]	5.55 [4.62, 8.05]	< 0.001
4	Lactate (mmol/L)	60.34	1.10 [0.80, 1.50]	2.10 [1.40, 3.20]	5.80 [3.70, 9.30]	< 0.001
5	White blood cells (× 10^9^/L)	13.05	12.85 [9.67, 18.90]	13.85 [7.15, 20.83]	6.80 [2.30, 19.20]	0.085
6	Platelet count (× 10^9^/L)	20.57	233.0 [176.25, 296.50]	168.50 [111.0, 231.0]	101.5 [52.75, 158.5]	< 0.001
7	HCO_3_ (mmol/L)	29.53	24.00 [22.00, 30.00]	19.00 [16.00, 22.00]	15.00 [12.00, 18.00]	< 0.001
8	Median creatinine (µmol/L)	9.09	70.00 [51.00, 104.5]	118.5 [75.75, 220.5]	191.0 [104.0, 256.0]	< 0.001
Comorbidity						
9	Nonpulmonary sepsis	3.05	51.0 (79.7%)	45.0 (52.3%)	14.0 (46.7%)	0.001
10	Non-cardiogenic shock	4.89	14.0 (21.9%)	43.0 (50.0%)	19.0 (63.3%)	< 0.001
11	Sepsis	4.24	46.0 (71.9%)	79.0 (91.9%)	29.0 (96.7%)	< 0.001
Biomarker						
12	Protein C (IU/ml)	14.23	87.56 [63.66, 120.8]	58.34 [42.29, 76.36]	45.88 [29.72, 59.02]	< 0.001
13	IL-6 (pg/ml)	83.85	31.85 [12.98, 58.67]	215.9 [65.97, 541.76]	1662.95 [1464.12, 1703.72]	< 0.001
14	IL-8 (pg/ml)	92.80	17.52 [11.78, 25.85]	89.53 [42.91, 245.76]	1309.68 [415.08, 1876.56]	< 0.001
15	IL-10 (pg/ml)	74.51	1.87 [0.97, 2.60]	8.73 [5.73, 17.89]	95.00 [47.02, 237.14]	< 0.001
16	TNF-α (pg/ml)	80.75	4.32 [3.01, 5.58]	9.34 [5.81, 17.86]	60.86 [37.06, 86.10]	< 0.001
17	ANG-2 (ng/ml)	26.00	15599.28 [9121.07, 22569.53]	27227.89 [16780.04, 57219.49]	66847.94 [48797.5, 119579.71]	< 0.001
18	RAGE (pg/ml)	12.80	602.97 [347.1, 1167.91]	1230.46 [585.74, 2943.99]	3391.97 [1323.99, 6817.59]	< 0.001
19	vWF (IU/ml)	8.89	241,782.09 [83908.68, 548,187.40]	663,133.03 [286863.6, 1538970.55]	1457472.55 [838028.35, 5753542.29]	< 0.001
20	TNF-R1 (ng/ml)	5.89	7304.35 [5534.83, 11,361.04]	19851.10 [12101.96, 28226.78]	31,518.88 [20929.46, 47527.00]	< 0.001
21	ICAM-1 (ng/ml)	17.25	623603.80 [472636.42, 834363.55]	816805.24[608061.95, 1138757.95]	1588277.33 [924274.89, 2198940.42]	< 0.001
22	PAI-1 (ng/ml)	56.91	45654.30 [28980.94, 76908.59]	144004.81 [84677.94, 345661.74]	920145.05 [541715.8, 1436526.94]	< 0.001
23	SPD (ng/ml)	NA	5100.07 [2468.15, 8446.50]	2601.78 [1410.88, 6223.18]	2388.34 [1091.56, 3259.15]	0.001
Endpoint						
	Death at ICU Discharge		0 (0%)	25 (29.4%)	19 (63.3%)	< 0.001

Data are reported by frequency and proportion (%) for discrete variables and median and interquartile range [Q1, Q3] for continuous variables

BMI body masse index, *PEEP* positive and expiratory pressure, *IL* interleukin, *TNF-α* tumour necrosis factor α, *ANG-2* Angiopoietin 2, *RAGE* receptor of advanced glycation end products, *vWF* von Willelbrand factor, *TNF-R1* tumour necrosis factor receptor 1, ICAM-1 intercellular adhesion molecule-1, *PAI-1* plasminogen activator inhibitor-1, *SPD* surfactant protein D and *Prot C* Protein C

We cross-classified our clustering results to the hyper- and hypo-inflammatory subphenotypes suggested by Sinha et al. (2020) [18] (Table S2). We observed 98–100% exact matches between Group 1 (mild) and hypo-inflammatory subtype and 100% between Group 3 (severe) and hyper-inflammatory subtype. For both models, 86 patients of Group 2 (moderate) are spread almost equaly between the hyper- and hypo-inflammatory groups (Table S2).

## Discussion

We simultaneously performed subgroup identification and variable selection using a single-step latent class model. Our work suggests that there are more than two ARDS subgroups. As has been reported in seminal studies, ARDS patients in this study appear to have an endotype that is either hypo-inflammatory (Endotype 1) or hyper-inflammatory (Endotypes 2 and 3) [15]. Our results are consistent with these findings but suggest that there may be a distinction in the hyper-inflammatory endotype identifying a group with a substantially higher mortality risk (Endotype 3). The separation between endotypes becomes evident by adding continuous variables (predominantly physiologic and biomarkers) to clinical binary variables (cause of shock, cause of ARDS, use of vasopressors) as discriminatory determinants in the model. For example, the hypo-inflammatory endotype has less vasopressor use, higher P/F ratios at baseline, lower serum lactate levels, and less evidence of kidney injury. Pro-inflammatory and anti-inflammatory marker differences between endotypes do not easily correspond with hypo- or hyper-inflammatory states. For example, IL-6 is higher in the hyperinflammatory endotype, yet IL-10 is much higher in at least the hyperinflammatory endotype three than the hypo-inflammatory endotype 1. Despite prior work by others identifying a role for RAGE—a biomarker relatively specific to lung epithelial injury, our results do not identify this biomarker in isolation as different between subgroups.

ARDS endotyping has undergone significant research over the last 10 years. Sequential findings have helped us understand how ARDS phenotypes can be detected and how they may be applied to different ARDS therapies. In 2014, Calfee et al. described the use of latent class methodology to identify two subphenotypes of ARDS, one of which was characterized as having more inflammation, shock, and metabolic acidosis, called Phenotype 2 (hyperinflammatory), and it was found to have a worse clinical outcome than Phenotype 1 (hypo-inflammatory) [15]. These phenotypes were derived from patient information from the ARMA trial (a trial of low vs high tidal volume). Three variables were found to differentiate these phenotypes (IL-6, sTNF-R1, and vasopressor use). Importantly, these phenotypes were validated and were found retrospectively to have a differential response to PEEP in patients from the ALVEOLI Trial (high vs low PEEP). In 2015, Calfee et al., using plasma biomarkers of lung epithelial and endothelial injury as well as inflammation, found two phenotypes characterized by evidence of direct lung injury (consistent with more epithelial lung injury and less severe endothelial lung injury), whereas indirect lung injury (consistent with more severe endothelial lung injury and less severe epithelial lung injury) suggestive of different molecular mechanisms of injury that may be used for potential future therapies [2]. Other studies have also examined mechanisms of injury in the direct and indirect lung injury phenotypes of ARDS using metabolomics and protein levels in serum [23]. In 2016, Famous et al. used patient information from the FACTT Trial (fluid and catheter treatment trial, a fluid management trial) to confirm two ARDS subphenotypes using latent class analysis (hyper- and hypo-inflammatory) that showed a differential response to fluid management [13]. They identified three variables that accurately classified the subphenotypes (IL-8, bicarbonate, and sTNF-R1) in keeping with previous studies. In 2017, Bos et al. used a different approach to examine ARDS subphenotypes [17]. They questioned whether plasma biomarkers (markers of inflammation, coagulation, and endothelial activation) alone could be used for subphenotype ARDS patients. They used cluster analysis and found two subphenotypes (uninflamed and reactive) based on four biomarkers (IL-6, interferon-gamma, angiopoietin 1/2, and PAI-1) with different mortality rates. They concluded that these two subpenotypes were similar to those previously identified as hyper-inflammatory and hypo-inflammatory.

To summarize and help apply the sub-phenotyping work, in 2020, Sinha et al. used patient information from 5 clinical trials (ARMA, ALVEOLI, FACTT, START, and HARP-2) to develop and validate a parsimonious classification model to accurately subphenotype ARDS meant to be used in the clinical setting [18]. They found a model having 3 or 4 variables (IL-8, bicarbonate, protein C, and vasopressor use) that could accurately identify the previously found two subphenotypes of ARDS (hyper-inflammatory and hypo-inflammatory). They propose that these markers could be used in ARDS clinical trials.

We compared our results to previously published models by cross-classifying our clustering results to the hyper- and hypo-inflammatory subphenotypes suggested by Sinha et al. (2020) [18] (Table S2). As presented in the result section, we observed 98–100% exact matches between Group 1 (mild) and the hypo-inflammatory subtype and 100% between Group 3 (severe) and the hyper-inflammatory subtype. For both models, 86 patients of Group 2 (moderate) are spread almost equaly between the hyper- and hypo-inflammatory subgroups. As a result of this comparison, we believe that our model, using more pathophysiologic biomarkers, allows more accurate grouping of patients supported by differential prediction of mortality between our two hyper-inflammatory groups. Moreover, if we apply the models of Sinha et al. (2020) [18] we have good accuracy and Kappa-agreement suggesting that our population are comparable (Table S2).

It is important to note that none of these investigators have said that only two subphenotypes exist, but their data model best fits two subphenotypes. Our data suggest a unique hyper-inflammatory subgroup may have a differential mortality risk. Although many prior studies of ARDS phenotyping have suggested only 2 clusters, other authors have also suggested that more than two phenotypes may exist [17].

Our study has numerous unique characteristics, strengths, and limitations. Our cohort of patients is unique to one region in Canada and may represent a more homogeneous cohort of patients within a complex heterogeneous disease than those previously described. For example, in the Calgary region, there are fewer African Americans and Hispanic heritage people, and there are more people of Asian heritage and Indigenous individuals than seen in most U.S. centers. Of note, we did not get information on heritage in our study. In addition, although our patients arise from 2 physically distinct ICUs, the ICUs are from the same clinical department within which there is standardized training of respiratory therapists and ICU nurses, standardized equipment (e.g., monitors, ventilators, pulse oximeters, arterial blood gas analyzers, and intravenous pumps), a singular medication library, and medical staff, including critical care fellows, that rotate between units. Therefore, it is likely that care is comparable and homogeneous between ICUs. However, differences in race and ancestral origin may limit external generalizability. Race is not routinely collected by our electronic health medical record.

One strength of our study is that the cohort is an observational cohort rather than a highly selected patient sample, such as the prior studies that used patients enrolled in randomized controlled clinical trials, suggesting that these patients were highly selected relative to a population of interest. This can have introduced a selection bias in the population analyzed, as we all know that not all comers are eligible for those studies. As such, our cohort may more reasonably represent the 'usual' population of patients with ARDS, at least in Canada. Of note, our patients excluded those with COVID-19, which some have suggested have a unique or different endotype to other causes of ARDS.

Other strengths of our study include a standardized collection of clinical, laboratory, and biomarker data at admission. However, the levels of several biomarkers in our studies differed from those obtained in comparable studies. Notably, other more recent comparative work done with ARDS caused by COVID-19 has similar numbers [24].

Again, patients in other studies were enrolled in randomized controlled studies. This could result in a difference in the populations in those studies. Also, although blood samples were collected within a standardized protocol, including comparable times from ICU admission, samples were stored and batched until they were analyzed. Despite rigorous standardized operating procedures for processing and − 80℃ storage (including alarmed freezers) until analyzed, it is possible that biological material degradation occurred over time; this may have differentially affected our analyses. However, because of the uniformity of our standard operating procedures and sample management, it is less likely that there was sample degradation than that seen in all the randomized trials from many centers in the U.S. Those different results can also be attributed to technical differences, including the measurement and analysis methods—the kits used—for the biomarkers. In addition, most other studies collected blood for biomarkers at variable time points rather than standardized at admission, like in our study. In addition, other studies measured biomarkers at different times, either during the primary analysis of the initial study or during their analysis a few years later. Finally, it is important to notice a great difference in the biomarkers introduced in the models presented in the different studies. Most prior studies introduced more biomarkers in their models compared to our work, but the justifications for each biomarker selection in their model were not provided. Our study carefully chose the biomarkers in the clustering model; the biomarkers are also close to that of the most recent work published published [10, 11], and we used robust analytical techniques. Our findings also duplicate other recently published findings as described above [13, 15, 17, 25].

Our biomarkers did not include other potential markers, such as those from genomics or metabolomics: numerous studies have suggested that patients with ARDS may have unique genomic or metabolomic profiles [23], but one of our aims was to develop a method that could be relatively easily utilized in the ICUs in North America.

Our study used an advanced LCM technique compared to those working with a conventional LCM technique. Despite the efforts to include mixed-type data, small samples in high dimensions, and variable selection (which a conventional LCM cannot do), our study still has two analytical concerns about dependency between features. The first concern is that feature redundancy and dependency between feature groups still remain. For example, consider two groups of cytokine measures. IL-6, IL-8, IL-10, and TNFa are the markers of an inflammatory group, and ICAM-1, ANG-2, and vWF are endothelium group markers. One measure per group may be much more efficient when utilizing the model as a bedside formula. In addition, the model does not fully describe the association between markers within or between groups. The second concern is that equal weight is given to all variables included in a model. This assumption ignores pathobiological pathways, that is, pathways with individual effects on biomarkers (and vice versa), and the interdependent effects between biomarkers. Although our study demonstrates important cytokines or lab results that contribute to identifying phenotypes, it is impossible to attribute a weight of effect to each input feature.

## Conclusion

This study again highlights that patients with ARDS admitted to the ICU have heterogeneous characteristics and outcomes. Furthermore, simple characterization based on the P/F ratio alone may not be sufficient to estimate the risk of adverse outcomes such as mortality. Phenotyping studies have now been undertaken for several years. All the results of those studies are expected to be hypothesis-generating studies. In addition to other phenotyping studies, the present study also emphasizes the importance of undertaking new prospective studies with real-time measurements of biomarkers. Our study adds to the large body of evidence supporting that identifying unique endotypes using rapid diagnostics measures including a limited biomarker profiles combined with clinical variables to impact clinical trial design or prognosticate patient outcomes, remains an unrealized opportunity rather than an intervention that can be implemented in real-time. Our study revealing three endotypes in ARDS is one of many studies that may advance the detection of ARDS endotypes in developing therapies or interventions in the future.

### Supplementary Information

Supplementary Material 1.

## Data Availability

The data sets used and/or analyzed during the current study are available from the corresponding author upon reasonable request.
