# Supplementary material for: Endotyping in ARDS: one step forward in precision medicine
Source: Eur J Med Res. 2024 May 14;29:284. doi: 10.1186/s40001-024-01876-7 (PMC11092098; doi:10.1186/s40001-024-01876-7)
Supplement: Supplementary file 1 — Supplementary Material 1. [file 40001_2024_1876_MOESM1_ESM.docx]

**Supplement**

Table S1. Information criteria for model selection and the number of patients found.

| # of groups | BIC | MICL | G1 | G2 | G3 | G4 | G5 |
| --- | --- | --- | --- | --- | --- | --- | --- |
| 1 | -10171.658 | -10256.833 | 180 |  |  |  |  |
| 2 | -9727.384 | -9837.650 | 91 | 89 |  |  |  |
| 3 | -9620.279 | -9767.354 | 86 | 30 | 64 |  |  |
| 4 | -9622.144 | -9785.518 | 54 | 49 | 47 | 30 |  |
| 5 | -9650.855 | -9818.872 | 29 | 18 | 47 | 43 | 43 |

Figure S1. Information criteria computed by a latent class model for model selection.


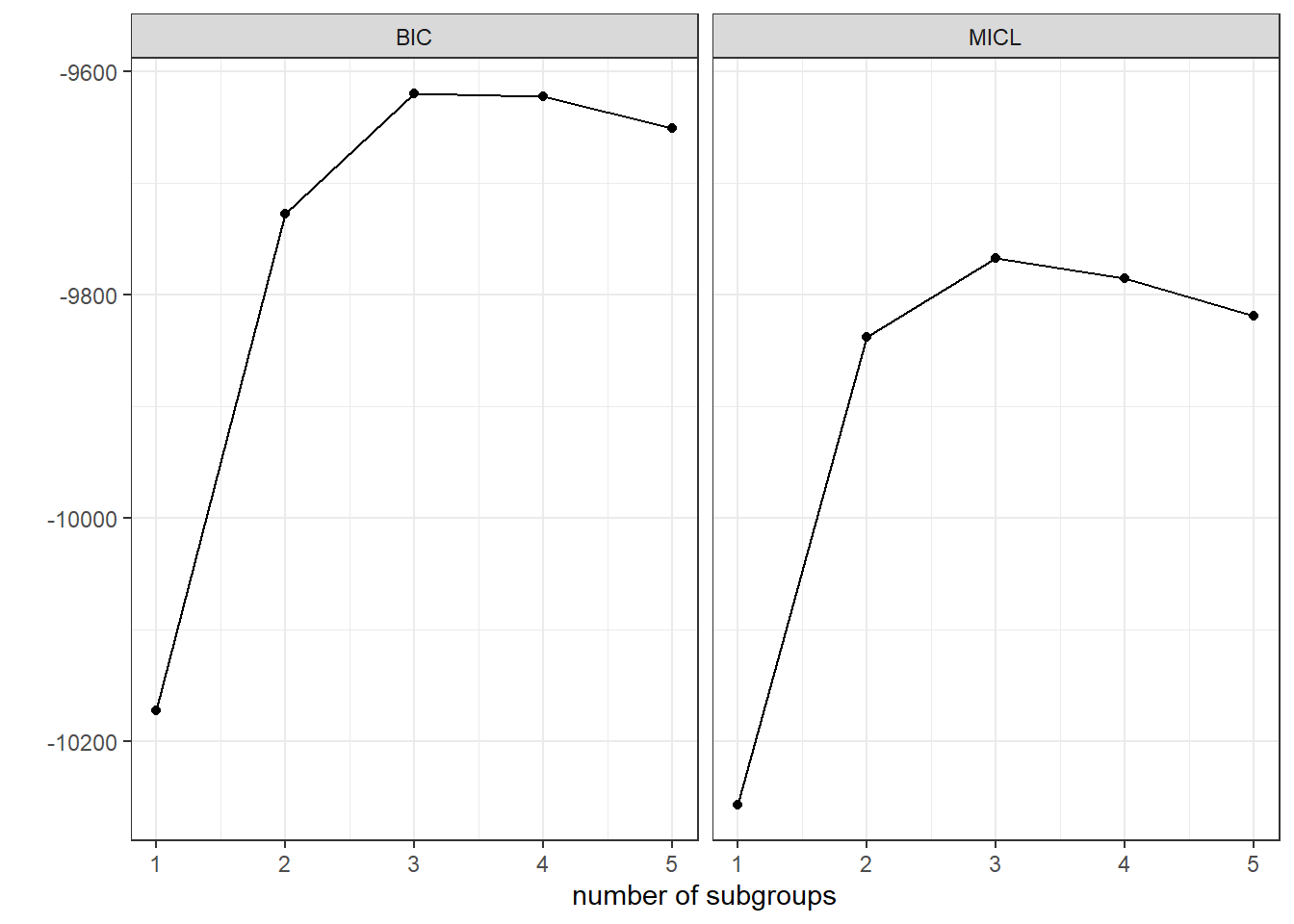


Table S2: Comparison of the model found in this study with the parsimonious model described by Sinha et al. in 2020. We utilized the following two formulas in Sinha et al. (2020).

Model 1: 6.1241 + 1.4226*log(IL-8+1) – 0.2596*Bicarbonate – 1.8330*log(protein-C+1)
Model 2: 4.8127 + 1.5424*log(IL-8+1) – 0.2502*Barcarbonate – 1.8778*log(protein-C+1) + 2.1026*Vasopressor

We cross-classified our clustering results to the hyper- and hypo-inflammatory subphenotypes classified. We observed 98%-100% exact matches between G1 (mild) and Hypo-inflammatory subtype and 100% between G3 (severe) and Hyper-inflammatory.

|  | Model 1 | | Model 2 | |  |
| --- | --- | --- | --- | --- | --- |
| LCA | Hyper | Hypo | Hyper | Hypo | Total |
| G1 (mild) | 0 | 64 | 1 | 63 | 64 |
| G2 (moderate) | 45 | 41 | 62 | 24 | 86 |
| G3 (severe) | 30 | 0 | 30 | 0 | 30 |

We also compared the subtypes found by two models. The accuracy is 0.8778 (95% CI: 0.8208, 0.918), and the kappa agreement is 0.7569.

|  |  | Model 2 | |  |
| --- | --- | --- | --- | --- |
|  |  | Hyper | Hypo | Total |
| Model 1 | Hyper | 73 | 2 | 75 |
|  | Hypo | 20 | 85 | 105 |
|  | Total | 93 | 87 | 180 |
